# Vocal fold fibroblasts and exposure to vibration *in vitro*: Does sex matter?

**DOI:** 10.1371/journal.pone.0297168

**Published:** 2024-02-09

**Authors:** Andrijana Kirsch, Tanja Grossmann, Barbara Steffan, Andrea Groselj-Strele, Claus Gerstenberger, Markus Gugatschka

**Affiliations:** 1 Division of Phoniatrics, ENT University Hospital, Medical University of Graz, Graz, Austria; 2 Core Facility Computational Bioanalytics, Center for Medical Research, Medical University of Graz, Graz, Austria; University of Vermont College of Medicine, UNITED STATES

## Abstract

Studies have shown that certain vocal fold pathologies are more common in one sex than the other. This is often explained by differences in the composition of the lamina propria and anatomical differences between female and male vocal folds, resulting in e.g. different fundamental frequencies. Here, we investigated a potential sex-specific voice frequency effect in an *in vitro* setting using vocal fold fibroblasts from one male and one female donor with and without cigarette smoke extract (CSE) addition. After exposure to either male or female vibration frequency with or without CSE, cells and supernatants were harvested. Gene and protein analysis were performed by means of qPCR, western blot, ELISA and Luminex. We found that exposure of cells to both male and female vibration pattern did not elicit significant changes in the expression of extracellular matrix-, inflammation-, and fibrosis-related genes, compared to control cells. The addition of CSE to vibration downregulated the gene expression of *COL1A1* in cells exposed to the female vibration pattern, as well as induced *MMP1* and *PTGS2* in cells exposed to both female and male vibration pattern. The protein expression of MMP1 and COX2 was found to be significantly upregulated only in cells exposed to CSE and female vibration pattern. To conclude, different vibration patterns alone did not cause different responses of the cells. However, the female vibration pattern in combination with CSE had a tendency to elicit/maintain more pro-inflammatory responses in cells than the male vibration pattern.

## Introduction

Voice disorders affect almost 30% of the general population during their lifetime [1]. Several studies have shown that vocal fold (VF) pathologies occur more often in women than men, with some pathologies being more common in one gender [2, 3]. Likewise, a disproportionately higher rate of benign VF lesions is observed in women compared to men [4]. This susceptibility to phonotraumatic injury in women is often explained by structural differences in male and female laryngeal anatomy. Female larynges have shorter VF, producing voice at a higher fundamental frequency. As a consequence, there is less tissue mass to absorb a larger amount of vibratory force [1]. Additionally, female VF contain less hyaluronic acid (HA) in the superficial layer of the lamina propria, which would make the VF more prone to injury, as HA plays an important role in wound healing [5]. Vocal fold fibroblasts (VFF) are the most abundant cell type within the extracellular matrix (ECM) of the VF lamina propria and are key regulators of the ECM homeostasis, ensuring normal tissue architecture and function. Moreover, VFF are involved in wound healing upon VF injury, and play an important role in various VF pathologies [6–8]. It has been shown that vibrational biomechanical strain can increase VFF proliferation [9] and reduce inflammation after injury [10, 11]. However, a potential sex-specific voice frequency effect has not been investigated so far. As differences between male and female patients are clinically evident, we sought to address this question on the cellular basis.

Using a phonomimetic device [12], we exposed immortalized human VFF to vibration with or without a common noxious stimulus (cigarette smoke extract (CSE)). We applied either a typical male (100–135 Hz), or a typical female (200–250 Hz) frequency range/pattern, to determine whether the difference in stimulation frequency itself elicits different responses in the VFF in terms of ECM component production and inflammatory mediators. Since primary human VFF are difficult to obtain, two immortalized VFF cell lines, of female and male origin, were used for the study.

## Materials and methods

### Bioreactor settings

Two bioreactors [12] were used in parallel for the experiments, both using the same vibration pattern per experiment. The frequency ranges of the generated patterns were designed to include the fundamental frequencies of the male and female voice [13] (100–135 Hz and 200–250 Hz, respectively). The vibration pattern sound files were created using Audacity software (version 2.2.2, audacityteam.org, registered trademark of Dominic Mazzoni). Spectrums of membrane displacements of BioFlex® plates (Flexcell International Corporation, Burlington, NC, US) were measured using laser Doppler vibrometry as previously described [12].

### Cell culture

Immortalized human VFF [14] were kindly provided by Prof. Susan Thibeault, University of Wisconsin-Madison. Cells from both male (A8, age 21, RRID:CVCL_A4GG) and female (E7, age 59, RRID:CVCL_A4GJ) donors were used for the experiments. Cells were cultured at 37˚C with 5% CO_2_ in high glucose Dulbecco’s modified Eagle’s medium (DMEM, Sigma Aldrich, Vienna, AT) supplemented with 10% fetal bovine serum (FBS, Biowest, Nuaillé, FR) and 100 μg/ml Normocin (Invivogen, San Diego, CA, USA). Cells were trypsinized using 0.25% Trypsin/EDTA (Sigma Aldrich) and seeded on the flexible culture plates coated with pronectin at a density of 144 000 cells/well (A8) and 150 000 cells/well (E7), each in 3 wells/plate. E7 cells have a slightly different morphology, compared to A8 cells, therefore different cell numbers were used to achieve a similar cell confluency. Cigarette smoke extract (CSE) and air bubbled control (ABC) were prepared as previously described [15]. Cells were allowed to attach under static conditions for 24 hours, after which the medium was changed to DMEM containing 1% FBS and either 5% CSE or 5% ABC. Cells were then transferred to the vibration bioreactor for 72 hours. Cells were exposed to the vibration pattern as followed: 8 hours without vibration (rest) followed by 16 hours composed of 30 seconds vibration (either male or female vibration pattern) and 90 seconds rest (total of 4 hours vibration per day). Non-vibrational control cells were cultivated in parallel in a separate incubator. After 72 hours, cells were harvested and medium was collected for subsequent analysis. Six experiments were performed for each vibration pattern.

### Analyses

#### LDH assay

Quantification of cytotoxicity was performed with cell culture supernatants using the Pierce LDH Cytotoxicity Assay Kit (Thermo Fisher Scientific, Waltham, MA, USA) as previously described [12], according to the manufacturer’s instructions. LDH activity is expressed as percentage of maximal LDH activity.

#### RNA isolation and RT-qPCR

RNA isolation was performed as previously described [12] using QIAZOL Lysis Reagent and miRNeasy Mini Kit (both from Qiagen, Hilden, Germany). Purified RNA was eluated in RNase-free water and concentration was determined using the NanoDrop 2000c spectrophotometer (Thermo Scientific). Reverse transcription (RT) was performed as described previously [6]. RT quantitative PCR (RT-qPCR) was performed using the Bio-Rad CFX 384 real-time PCR Detection System (Bio-Rad, Hercules, CA, USA) and quantified on CFX Maestro 1.1 Software (version 4.1.2433.1219, Bio-Rad). Self-designed primers were validated and melting curves were generated for all qPCR runs. For detection of the cDNA of one gene of interest, all samples were run on the same qPCR plate. Primer sequences are provided in Table 1. C_q_ values of technical triplicates were averaged and relative quantification of all mRNAs of interest was performed based on the 2^-ΔΔCq^ method [16] with a minor modification, the geometric mean of the C_q_ values of B2M and UXT reference RNAs was used as an internal normalization factor.

**Table 1:** Primer sequences used for RT-qPCR.

Gene	Full name	Forward primer	Reverse primer
*ACTA2*	Actin alpha 2	GTGGGTGACGAAGCACAGAG	GAGTGGTGCCAGATCTTTTCCA
*B2M*	Beta 2 microglobulin	AGGCTATCCAGCGTACTCCA	CGGATGGATGAAACCCAGACA
*COL1A1*	Collagen type I alpha 1	CCAAGACGAAGACATCCCACC	GTTTCCACACGTCTCGGTCA
*COL1A2*	Collagen type I alpha 2	ACCACAGGGTGTTCAAGGTG	CAGGACCAGGGAGACCAAAC
*COL3A1*	Collagen type III alpha 1	GAGGATGGTTGCACGAAACAC	GGTAGTCTCACAGCCTTGCG
*FN1*	Fibronectin	CTGCAAGCCCATAGCTGAGA	GAAGTGCAAGTGATGCGTCC
*HAS2*	Hyaluronan synthase 2	ATGCTTGACCCAGCCTCATC	TTAAAATCTGGACATCTCCCCCAA
*HAS3*	Hyaluronan synthase 3	GACTACATCCAGGTGTGCGA	ATCCTCCTCCAGGACTCGAA
*HYAL1*	Hyaluronidase 1	GCCCTTGCACTCCTACTGAG	ATTTGCTGGACTGGTGCCTC
*HYAL2*	Hyaluronidase 2	CCACAAGCACGGAGACCTG	CCCAGGACACATTGACCACG
*IL6*	Interleukin 6	AACCCCCAATAAATATAGGACTGGA	CCGAAGGCGCTTGTGGA
*MMP1*	Matrix metallopeptidase 1	CACGCCAGATTTGCCAAGAG	GTTGTCCCGATGATCTCCCC
*PTGS2*	Cyclooxygenase 2	AGAAAACTGCTCAACACCGGAA	TGCACTGTGTTTGGAGTGGG
*TGFB1*	Transforming growth factor beta 1	TACCTGAACCCGTGTTGCTC	GCTGAGGTATCGCCAGGAAT
*UXT*	Ubiquitously expressed prefoldin like chaperone	GCAGCGGGACTTGCGA	TAGCTTCCTGGAGTCGCTCA
*VEGFA*	Vascular endothelial growth factor A	GGCAGAATCATCACGAAGTGG	GGCACACAGGATGGCTTGA

#### Western blot

Cells were washed twice with ice-cold phosphate-buffered saline (PBS) and lysed in RIPA buffer (Cell Biolabs, San Diego, CA, USA) supplemented with 1x Halt Protease and Phosphatase Inhibitor Cocktail and 5 mM EDTA (both Thermo Fisher Scientific). Protein content was determined using Pierce BCA Protein Assay Kit (Thermo Fisher Scientific) according to the manufacturer’s instructions. Fifteen μg of total protein was mixed with appropriate amounts of 4x Laemmli Buffer (Bio- Rad, Hercules, CA, USA) and dithiothreitol (DTT) and boiled for 5 minutes at 95 ˚C. SDS-PAGE was performed using 4–20% Mini PROTEAN TGX gels (Bio-Rad), after which the proteins were blotted onto a polyvinylidene fluoride (PVDF) membrane (Bio-Rad). The blots were blocked in 5% milk for 2 hours, followed by incubation with the primary antibody over night at 4 ˚C (COL1A1: Nordic Bio Site, AB_2892675; COX2: Thermo Fisher Scientific; AB_2533224; GAPDH: Cell Signaling Technology, AB_561053; MMP1: Proteintech, AB_2297741). After washing, the blots were incubated with the appropriate HRP-conjugated secondary antibody (Agilent Dako, AB_2617137 and AB_2617138). Bands were detected after the addition of SuperSignal West Pico Chemiluminescent Substrate (Thermo Fisher Scientific). Blot images were acquired with the ChemiDoc Touch system (Bio-Rad) and densitometric analysis was performed using ImageLab software (Bio-Rad). After detection, the blots were stripped for 10 minutes using Restore PLUS western blot stripping buffer (Thermo Fisher Scientific), and detection of further proteins was performed as indicated above.

#### Magnetic Luminex® Assay

Proteins of interest were determined in supernatants using custom Human High Sensitivity Cytokine A Premixed Magnetic Luminex Performance Assay (Biotechne, R&D Biosystems) for analysis of VEGFA according to manufacturer’s instructions. Standard curves were generated from provided analyte standards. Sample dilution, determined from previous experiments, was 1:4. The assay was measured on the Bio-Plex 200 assay reader and concentrations were calculated using the Bio-Plex Manager Software, version 6.2 (both Bio-Rad).

#### Hyaluronan ELISA

Hyaluronan (HA) content was measured in supernatants (1:40 dilution) using the Quantikine ELISA (R&D Systems, Abingdon, UK), according to the manufacturer’s instructions.

#### Statistical analysis

Shapiro-Wilk test was used to assess the normal distribution of the data. Due to the experimental setup, the statistical analysis was performed in two steps. First, for each vibration pattern (male or female) and cell line, a repeated measures (RM) one-way ANOVA with Šídák multiple comparison test or Friedman test with Dunn’s multiple comparisons test was used to test for differences between vibration, combination of CSE and vibration, and static control. In a second step, the data was normalized as fold change compared to control, and a two-way ANOVA followed by Tukey’s multiple comparison test was used to compare the effect of different vibration patterns and cell lines. P values below 0.05 were considered statistically significant. All statistical analyses were performed using GraphPad Prism version 9.0 (San Diego, CA, USA).

## Results

**Fig 1:**
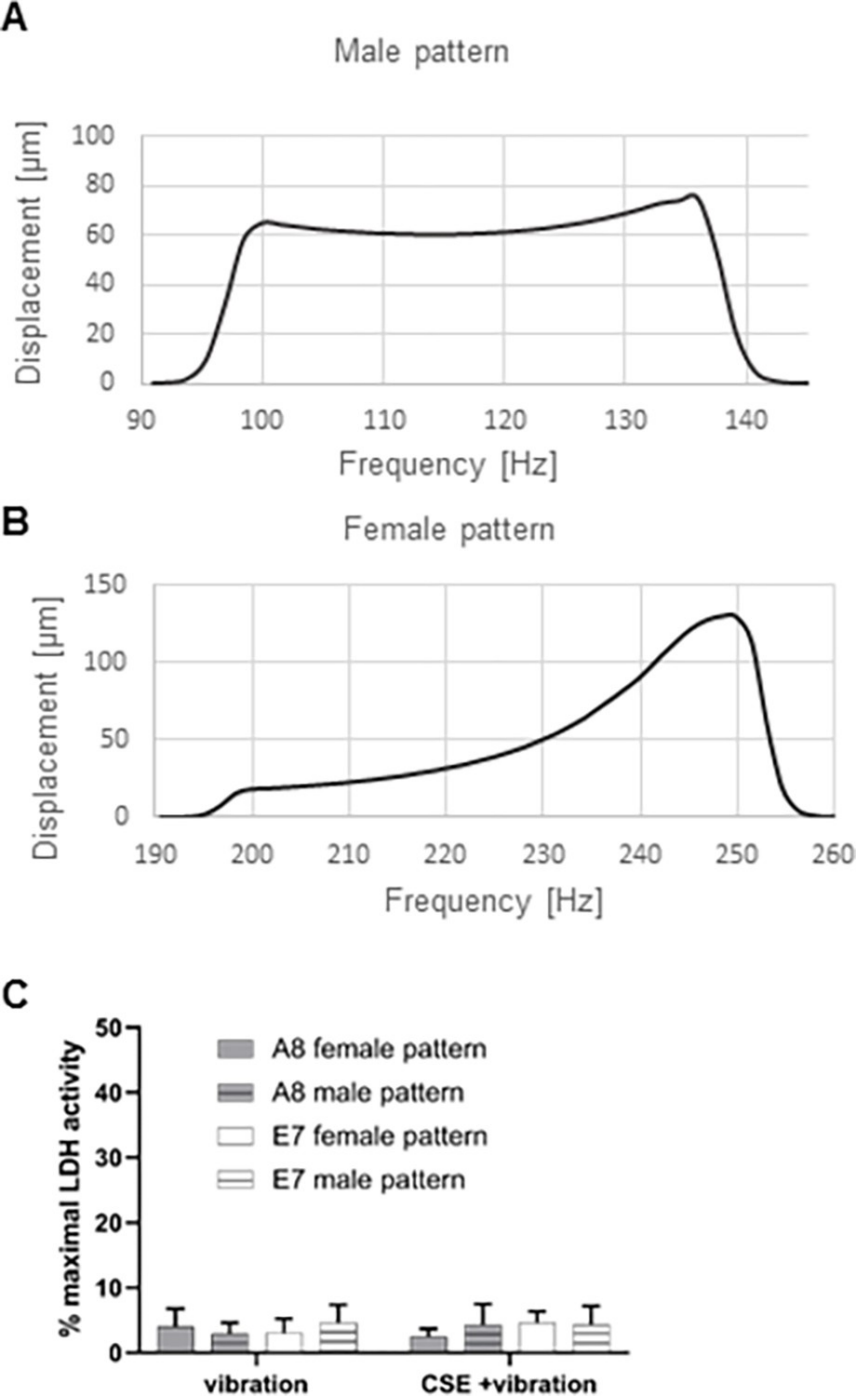
Membrane displacements and cytotoxicity. Spectrum of membrane displacement in the Bioflex 6-well culture plates when stimulated in the fundamental frequency range of the male (A) and female (B) voice. LDH activity assay shows no cytotoxicity induced by the treatment (C). Membrane displacement data are shown as median from 6 wells of a plate; measured at the center of the membranes. LDH assay data are shown as mean with SD of six independent experiments (N = 6).

The male and female vibration pattern induced membrane displacements of 60–80 μm and 20–130 μm, as shown in Fig 1A and 1B, respectively. The LDH activity assay revealed that exposure of cells to vibration alone, or in combination with CSE was not cytotoxic (Fig 1C).

**Fig 2:**
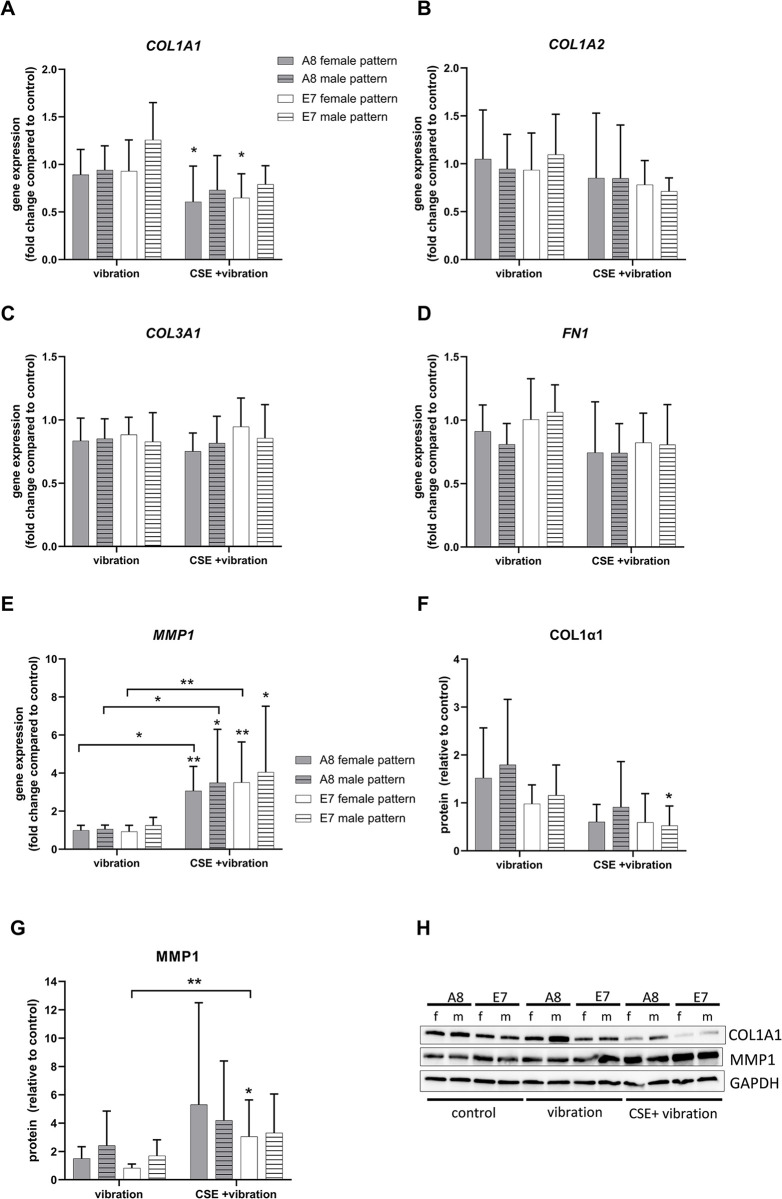
Effect of vibration and CSE on the ECM. Gene expression levels of ECM-related genes *COL1A1* (A), COL1A2 (B), *COL3A1* (C), *FN1* (D) and *MMP1* (E) were analyzed by RT-qPCR and protein levels of COL1α1 (F) and MMP1 (G) were analyzed by Western blot. Representative blots of COL1α1 and MMP1 are shown in (H). All data are shown as mean and SD of fold changes compared to static control from six independent experiments (N = 6). Differences between the treatments within one vibration pattern and cell line were tested using RM one-way ANOVA with Šídák multiple comparison test or Friedman test with Dunn’s multiple comparisons test, depending on the result of the Shapiro-Wilk test of normality. Two-way ANOVA followed by Tukey’s multiple comparison test was used to compare the effect of different vibration patterns and cell lines. Unless otherwise indicated, asterisks show difference to static control, * *P* < 0.05, ** *P* < 0.01. f = female vibration pattern, m = male vibration pattern.

Gene expression analysis of ECM-related genes showed no statistically significant effect of vibration on the expression of collagen (*COL1A1*, *COL1A2* and *COL3A1*), fibronectin (FN1) and matrix metallopeptidase 1 (MMP1) expression (Fig 2A–2E, respectively). However, the addition of CSE significantly reduced the gene expression of *COL1A* in both cell lines exposed to the female pattern. The gene expression of *MMP1* was significantly upregulated with CSE addition in all conditions compared to control. The protein expression of COL1α1 tended to be downregulated with the addition of CSE to vibration, but was significantly downregulated only in E7 cells exposed to male vibration and CSE (Fig 2F and 2H). MMP1 protein expression was significantly upregulated in E7 cell line samples exposed to the female pattern and CSE, compared to control, although a trend toward upregulation was seen for all cell lines and vibration patterns in combination with CSE (Fig 2G and 2H). Protein levels of COL1α2, Col3α1 and FN1 were not significantly altered (S1 Fig).

**Fig 3:**
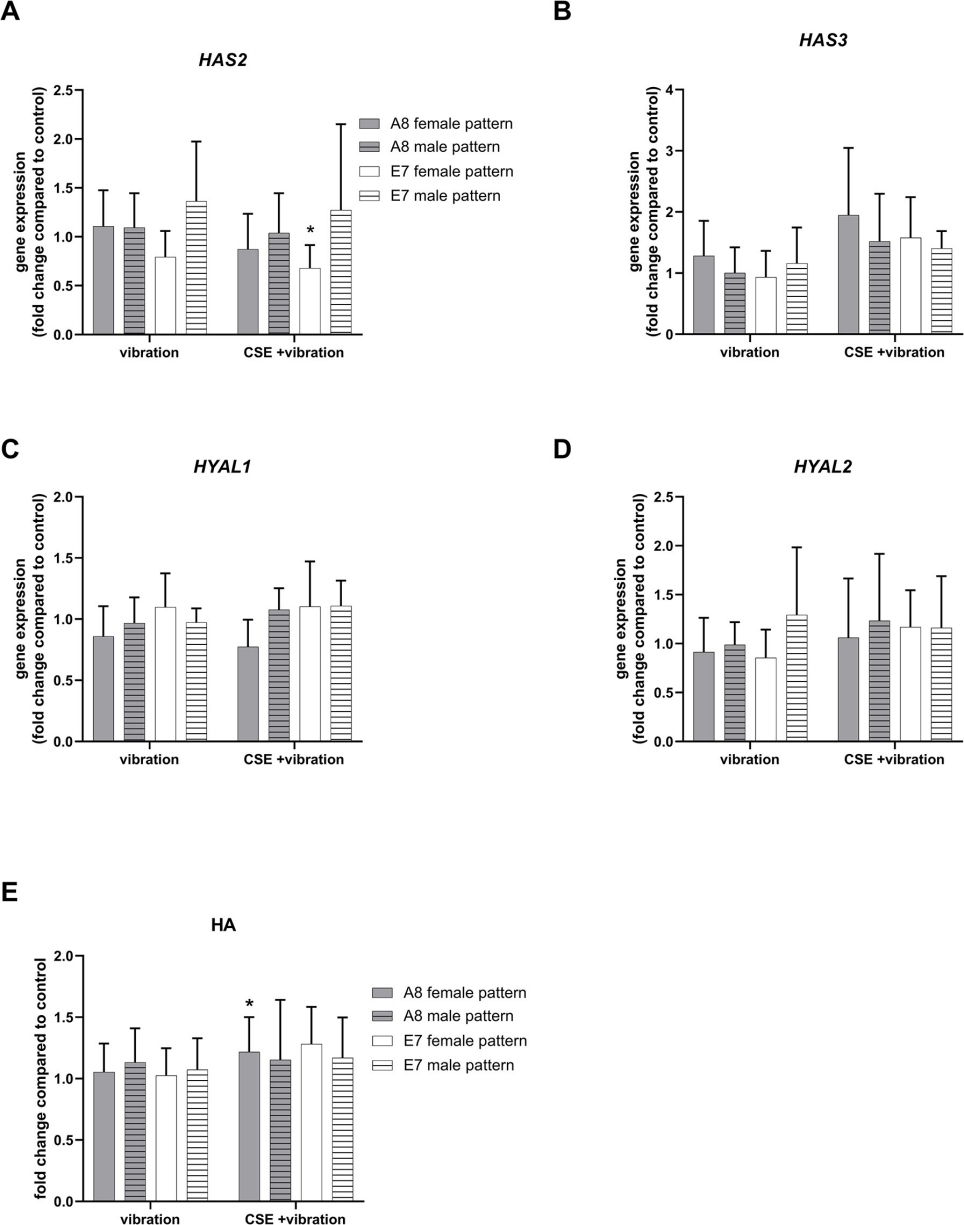
Effect of vibration and CSE on the HA metabolism. Gene expression levels of HA metabolism-related genes HAS2 (A), HAS3 (B), HYAL1 (C) and HYAL2 (D) were analyzed by RT-PCR. Total HA content in supernatants was analyzed with ELISA (E). All data are shown as mean and SD of fold changes compared to static control from six independent experiments (N = 6). Differences between the treatments within one vibration pattern and cell line were tested using RM one-way ANOVA with Šídák multiple comparison test or Friedman test with Dunn’s multiple comparisons test, depending on the result of the Shapiro-Wilk test of normality. Two-way ANOVA followed by Tukey’s multiple comparison test was used to compare the effect of different vibration patterns and cell lines. Unless otherwise indicated, asterisks show difference to static control, * *P* < 0.05.

Gene expression of HA metabolism genes was not affected by vibration (Fig 3A–3D). However, the combination of CSE and female vibration pattern significantly reduced the gene expression of HA synthase 2 (*HAS2*) in the E7 cell line, compared to the control. HA levels measured by ELISA were slightly but significantly elevated in A8 cells exposed to CSE and female vibration pattern (Fig 3E).

**Fig 4:**
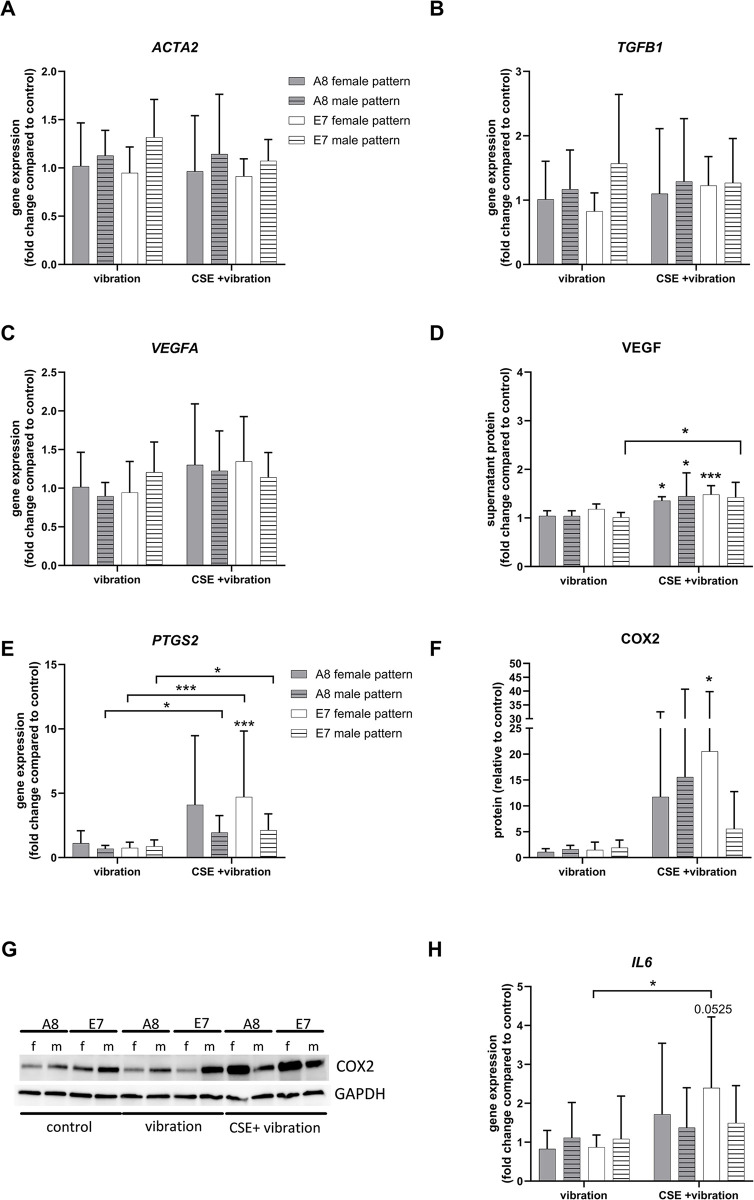
Fibrosis, angiogenesis and inflammation. Gene expression levels of fibrosis-related genes *ACTA2* (A) and *TGFB* (B) and angiogenesis-associated genes *VEGFA* (C) were analyzed by RT-qPCR. Protein expression of VEGF (D) was measured by Western blot. Gene expression levels of inflammation-associated *PTGS2* (COX2) and *IL6* were analyzed by RT-qPCR (E and H, respectively) and protein levels of. COX2 were analyzed by Western blot (F). A representative COX2 blot is shown in (G). All data are shown as mean and SD of fold changes compared to static control from six independent experiments (N = 6). Differences between the treatments within one vibration pattern and cell line were tested using RM one-way ANOVA with Šídák multiple comparison test or Friedman test with Dunn’s multiple comparisons test, depending on the result of the Shapiro-Wilk test of normality. Two-way ANOVA followed by Tukey’s multiple comparison test was used to compare the effect of different vibration patterns and cell lines. Unless otherwise indicated, asterisks show difference to static control, * *P* < 0.05, *** *P* < 0.001. f = female vibration pattern, m = male vibration pattern.

Both vibration alone and the combination of CSE and vibration did not affect the fibrosis-related genes actin alpha 2 (*ACTA2*) and transforming growth factor beta 1 (*TGFB1*) (Fig 4A and 4B, respectively). Vascular endothelial growth factor A (*VEGFA*), a factor that promotes angiogenesis was not significantly altered at the gene expression level (Fig 4C). However, a small, but significant upregulation at the protein level was measured in the supernatant of cells exposed to CSE in combination with vibration (Fig 4D). The expression of cyclooxygenase 2 (*PTGS2*, COX2, Fig 4E), an inflammation associated gene, was significantly upregulated only with the addition of CSE to vibration in E7 cells exposed to the female vibration patterns, compared to control, while a significant difference between vibration only and vibration and CSE was observed with the male vibration pattern for both cell lines. On the protein level, COX2 was significantly upregulated only in the E7 cell line exposed to the female vibration pattern and CSE, although a trend was seen for all cell lines and vibration patterns combined with CSE exposure (Fig 4F and 4G). The gene expression of interleukin 6 (*IL6)*, was not significantly upregulated with vibration or the combination of CSE and vibration, compared to control. However, a significant difference was observed in the E7 cell line exposed to the female pattern when comparing cells exposed to the vibration alone and the combination of vibration and CSE (Fig 4H).

## Discussion

Sex-specific differences in voice frequency are biologically and anatomically defined, as males have larger larynges and longer VF, compared to females [17], but the cellular effect of different frequency ranges cannot be studied *in vivo*, which is why we used an *in vitro* setting. The VFs are physiologically exposed to high frequency forces, thus the exposure to vibrational force in VF research is important in order to mimic those conditions *in vitro*. However, different studies used different vibration frequencies, ranging from 50–300 Hz, with most using a typical male frequency of 100 Hz [9, 12, 18–23]. As sex specific-differences in research, including basic research, have become more relevant, we wanted to answer the question if simply exposing VFF to sex specific vibration frequencies would evoke a different response of the cells. A positive answer might indicate that sex specific findings at the level of the VF (e.g. prevalence of VF polyps) have their origin starting at the cellular level. As a consequence, vibration frequency would need to be taken into consideration when conducting *in vitro* VF research.

The used female and male frequency patterns caused different membrane deflection ranges of the BioFlex® cell culture dish wells, however, a similar average deflection of approximately 60μm was achieved. We showed that a moderate exposure to vibration alone (male or female pattern, 4 hours per day) did not statistically alter the expression of ECM-, inflammation-, and fibrosis-related genes, compared to control cells. This is in line with a previous publication in which cells were exposed to the same amount of vibration per day, with the difference being the vibration frequency pattern, using a range of 50–250 Hz [11]. The addition of CSE to vibration, however, induced changes in the expression of several genes and proteins. A previous publication by Gugatschka et al. showed that CSE alone can cause a downregulation of *COL1A1* gene expression in VFF [15]. Here we observed that the combination of CSE and vibration downregulated *COL1A1* by 20–27% in cells exposed to the male vibration pattern, and by 35–40% in cells exposed to the female vibration pattern, compared to control. Only the downregulation in cells exposed to the female vibration pattern was statistically significant. The downregulation of Col1α1 was also observed on the protein level, although the combination of CSE and male pattern had the greatest effect. Therefore, we could not observe a clear impact of a specific vibration pattern. *MMP1* gene expression was upregulated by CSE and vibration, which was not surprising since *MMP1* has been reported to contain a promotor region responsive to CSE [24]. Different vibration patterns did not modulate the effect of CSE on *MMP1* expression. The upregulation of MMP1 protein compared to control, was also seen, and although similar mean values were observed for all patterns and cell lines, it was statistically significant only in the E7 cell line exposed to the combination of CSE and female vibration pattern. MMP1 digests interstitial collagen [25], therefore a reduction of Col1α1 in cells exposed to CSE and vibration is most likely a combination of gene downregulation and protein degradation. Female VF have less HA in the superficial layer of the lamina propria compared to male ones [26]. Although we observed a significant decrease of *HAS2* gene expression in E7 cells exposed to female vibration pattern and CSE, this did not affect the level of HA measured in the supernatant. A trend toward an increase in *HAS3* gene expression was observed in cells exposed to CSE and vibration, which, although not significant, may have contributed to HA production, as a small, but significant increase in HA production was observed in A8 cells exposed to the female vibration pattern and CSE. VEGF is involved in angiogenesis, inflammation, wound healing and tissue remodeling in the skin and airway [27–29]. Vibration alone had no effect on both the gene and protein expression, however the addition of CSE caused a slight but significant increase of VEGF protein measured in the supernatant for almost all vibration patterns and cell lines. This is in line with a previous publication by Grossmann et al. where fibroblasts exposed to CSE and vibration showed an increased VEGF protein in the supernatant, compared to control [30]. CSE can induce COX2 [30–33], leading to a pro-inflammatory environment. We found that the gene expression of *PTGS2* was significantly upregulated only in the E7 cell line exposed to the female pattern and CSE, although a trend was observed for all cell lines and patterns. This was also confirmed on the protein level. The gene expression of the pro-inflammatory *IL6* is also known to be upregulated by CSE [34]. We observed the highest *IL6* levels again in the E7 cell line exposed to the female pattern and CSE, compared to control, but missed statistical significance.

It is possible that the female vibration pattern exerted more tensile stress on the cells, due to higher maximum membrane displacements, compared to the male pattern (120 μm vs 80 μm, respectively). This, in combination with CSE, caused a significant change in the expression of the ECM-related genes, as well as MMP1 and COX2 protein, compared to the control. Moreover, the E7 cell line was derived from an older donor compared to the A8 cell line, which could have an impact on the inflammatory response of the E7 cell line. Aging is linked with an increase in inflammation [35, 36]. Thus, the E7 cell line may have epigenetic alterations that contribute to a stronger inflammatory response despite the immortalization, as a study suggests that hTERT immortalization does not prevent epigenetic aging [37].

At the time of immortalization, the karyotypes of used A8 and E7 cell lines contained XY and XX chromosomes [14], respectively. However, one must address the sex of immortalized cells with caution, since losses of Y chromosomes have been seen with higher passages in other cell lines [38]. Therefore, the cells used in this study are only considered as different VFF cell lines, not as biologically female or male cells. Sex hormones play a role in ear, nose, and throat diseases [39]. While there are immunohistochemical studies showing the expression and distribution of estrogen and androgen receptors in human vocal folds [40–42], one study claims that these are unspecific staining patterns and thus false positives [43]. Moreover, to the best of our knowledge, *in vitro* studies investigating the role of sex hormones on isolated VFF were done only using rat cells [44, 45]. As it is unclear if human VFF express steroid hormone receptors, we decided to omit the additional exposure of cells to different sex hormones.

In conclusion, moderate *in vitro* exposure to female and male vibration patterns alone had no significant effect on gene and protein expression of immortalized VFF. However, the addition of a noxious stimuli (CSE), did evoke certain differences in the effect of the male and female frequency pattern on the cells and should be investigated further. Therefore, the differences in VF pathologies are most likely not caused by the different vibration frequencies alone, and are rather a consequence of anatomical differences combined with external stimuli.

## Supporting information

S1 Fig Effect of vibration and CSE on ECM-related protein expression. Protein levels of COL1A2 (A), COL3A1 (B), and FN1 (D) were analyzed by Western blot (A, B) and measured in the supernatant (D). Representative blots of COL1α2 and COL3α1 are shown in (C). All data are shown as mean and SD of fold changes compared to static control from six independent experiments (N = 6). Differences between the treatments within one vibration pattern and cell line were tested using RM one-way ANOVA with Šídák multiple comparison test or Friedman test with Dunn’s multiple comparisons test, depending on the result of the Shapiro-Wilk test of normality. Two-way ANOVA followed by Tukey’s multiple comparison test was used to compare the effect of different vibration patterns and cell lines. f = female vibration pattern, m = male vibration pattern.
(TIF)

S1 Raw images Original uncropped and unadjusted Western blot images. (PDF)

S1 Raw data (XLSX)

## References

[1] Roy N, Merrill RM, Gray SD, Smith EM (2005) Voice disorders in the general population: prevalence, risk factors, and occupational impact. Laryngoscope 115(11):1988–1995. doi: 10.1097/01.mlg.0000179174.32345.41 16319611

[2] Coyle SM, Weinrich BD, Stemple JC (2001) Shifts in relative prevalence of laryngeal pathology in a treatment-seeking population. J Voice 15(3):424–440. doi: 10.1016/S0892-1997(01)00043-1 11575638

[3] Van Houtte E, Van Lierde K, D’Haeseleer E, Claeys S (2010) The prevalence of laryngeal pathology in a treatment-seeking population with dysphonia. Laryngoscope 120(2):306–312. doi: 10.1002/lary.20696 19957345

[4] Zhukhovitskaya A, Battaglia D, Khosla SM, Murry T, Sulica L (2015) Gender and age in benign vocal fold lesions. Laryngoscope 125(1):191–196. doi: 10.1002/lary.24911 25216037

[5] Ward PD, Thibeault SL, Gray SD (2002) Hyaluronic acid: its role in voice. J Voice 16(3):303–309. doi: 10.1016/s0892-1997(02)00101-7 12395982

[6] Grill M, Lazzeri I, Kirsch A, Steurer N, Grossmann T, Karbiener M, et al. (2021) Vocal Fold Fibroblasts in Reinke’s Edema Show Alterations Involved in Extracellular Matrix Production, Cytokine Response and Cell Cycle Control. Biomedicines 9(7). 10.3390/biomedicines9070735 PMC8301432 34206882

[7] Takeharu K, Kurakami K, Konomi U, Komazawa D, Misawa K, Imayoshi S, et al. (2018) Safety and short-term outcomes of basic fibroblast growth factor injection for sulcus vocalis. Acta Otolaryngol 138(11):1014–1019. doi: 10.1080/00016489.2018.1497808 30734621

[8] Ma Y, Long J, Amin MR, Branski RC, Damrose EJ, Sung CK, et al. (2020) Autologous fibroblasts for vocal scars and age-related atrophy: A randomized clinical trial. Laryngoscope 130(11):2650–2658. doi: 10.1002/lary.28453 31804729 PMC8136686

[9] Gaston J, Quinchia Rios B, Bartlett R, Berchtold C, Thibeault SL (2012) The response of vocal fold fibroblasts and mesenchymal stromal cells to vibration. PLoS One 7(2):e30965. doi: 10.1371/journal.pone.0030965 22359557 PMC3281043

[10] Branski RC, Perera P, Verdolini K, Rosen CA, Hebda PA, Agarwal S (2007) Dynamic biomechanical strain inhibits IL-1beta-induced inflammation in vocal fold fibroblasts. J Voice 21(6):651–660. doi: 10.1016/j.jvoice.2006.06.005 16905293 PMC4948979

[11] Hortobagyi D, Grossmann T, Tschernitz M, Grill M, Kirsch A, Gerstenberger C, et al. (2020) In vitro mechanical vibration down-regulates pro-inflammatory and pro-fibrotic signaling in human vocal fold fibroblasts. PLoS One 15(11):e0241901. doi: 10.1371/journal.pone.0241901 33211714 PMC7676657

[12] Kirsch A, Hortobagyi D, Stachl T, Karbiener M, Grossmann T, Gerstenberger C, et al. (2019) Development and validation of a novel phonomimetic bioreactor. PLoS One 14(3):e0213788. doi: 10.1371/journal.pone.0213788 30870529 PMC6417646

[13] Goy H, Fernandes DN, Pichora-Fuller MK, van Lieshout P (2013) Normative voice data for younger and older adults. J Voice 27(5):545–555. doi: 10.1016/j.jvoice.2013.03.002 23769007

[14] Chen X, Thibeault SL (2009) Novel isolation and biochemical characterization of immortalized fibroblasts for tissue engineering vocal fold lamina propria. Tissue Eng Part C Methods 15(2):201–212. doi: 10.1089/ten.tec.2008.0390 19108681 PMC2819707

[15] Gugatschka M, Darnhofer B, Grossmann T, Schittmayer M, Hortobagyi D, Kirsch A, et al. (2019) Proteomic Analysis of Vocal Fold Fibroblasts Exposed to Cigarette Smoke Extract: Exploring the Pathophysiology of Reinke’s Edema. Mol Cell Proteomics 18(8):1511–1525. doi: 10.1074/mcp.RA119.001272 31123107 PMC6683006

[16] Livak KJ, Schmittgen TD (2001) Analysis of relative gene expression data using real-time quantitative PCR and the 2(-Delta Delta C(T)) Method. Methods 25(4):402–408. doi: 10.1006/meth.2001.1262 11846609

[17] Titze IR (1989) Physiologic and acoustic differences between male and female voices. J Acoust Soc Am 85(4):1699–1707. doi: 10.1121/1.397959 2708686

[18] Titze IR, Hitchcock RW, Broadhead K, Webb K, Li W, Gray SD, et al. (2004) Design and validation of a bioreactor for engineering vocal fold tissues under combined tensile and vibrational stresses. J Biomech 37(10):1521–1529. doi: 10.1016/j.jbiomech.2004.01.007 15336927

[19] Kutty JK, Webb K (2010) Vibration stimulates vocal mucosa-like matrix expression by hydrogel-encapsulated fibroblasts. J Tissue Eng Regen Med 4(1):62–72. doi: 10.1002/term.219 19842110 PMC2849844

[20] Wolchok JC, Brokopp C, Underwood CJ, Tresco PA (2009) The effect of bioreactor induced vibrational stimulation on extracellular matrix production from human derived fibroblasts. Biomaterials 30(3):327–335. doi: 10.1016/j.biomaterials.2008.08.035 18937972

[21] Latifi N, Heris HK, Thomson SL, Taher R, Kazemirad S, Sheibani S, et al. (2016) A Flow Perfusion Bioreactor System for Vocal Fold Tissue Engineering Applications. Tissue Eng Part C Methods 22(9):823–838. doi: 10.1089/ten.tec.2016.0053 27537192 PMC5035918

[22] Farran AJ, Teller SS, Jia F, Clifton RJ, Duncan RL, Jia X (2013) Design and characterization of a dynamic vibrational culture system. J Tissue Eng Regen Med 7(3):213–225. doi: 10.1002/term.514 22095782 PMC4076702

[23] Kim D, Lim JY, Kwon S (2016) Development of Vibrational Culture Model Mimicking Vocal Fold Tissues. Ann Biomed Eng 44(10):3136–3143. doi: 10.1007/s10439-016-1587-5 26951463

[24] Mercer BA, Wallace AM, Brinckerhoff CE, D’Armiento JM (2009) Identification of a cigarette smoke-responsive region in the distal MMP-1 promoter. Am J Respir Cell Mol Biol 40(1):4–12. doi: 10.1165/rcmb.2007-0310OC 18617682 PMC2606945

[25] Pardo A, Selman M (2005) MMP-1: the elder of the family. Int J Biochem Cell Biol 37(2):283–288. doi: 10.1016/j.biocel.2004.06.017 15474975

[26] Butler JE, Hammond TH, Gray SD (2001) Gender-related differences of hyaluronic acid distribution in the human vocal fold. Laryngoscope 111(5):907–911. doi: 10.1097/00005537-200105000-00029 11359176

[27] Johnson KE, Wilgus TA (2014) Vascular Endothelial Growth Factor and Angiogenesis in the Regulation of Cutaneous Wound Repair. Adv Wound Care (New Rochelle) 3(10):647–661. 10.1089/wound.2013.0517 PMC4183920 25302139

[28] Lee KS, Park SJ, Kim SR, Min KH, Lee KY, Choe YH, et al. (2008) Inhibition of VEGF blocks TGF-beta1 production through a PI3K/Akt signalling pathway. Eur Respir J 31(3):523–531. doi: 10.1183/09031936.00125007 18057050

[29] Yuksel H, Yilmaz O, Karaman M, Bagriyanik HA, Firinci F, Kiray M, et al. (2013) Role of vascular endothelial growth factor antagonism on airway remodeling in asthma. Ann Allergy Asthma Immunol 110(3):150–155. doi: 10.1016/j.anai.2012.12.015 23548522

[30] Grossmann T, Steffan B, Kirsch A, Grill M, Gerstenberger C, Gugatschka M (2021) Exploring the Pathophysiology of Reinke’s Edema: The Cellular Impact of Cigarette Smoke and Vibration. Laryngoscope 131(2):E547–E554. doi: 10.1002/lary.28855 32569447 PMC7818424

[31] Steffan B, Grossmann T, Grill M, Kirsch A, Groselj-Strele A, Gugatschka M (2023) Comparing Effects of Short- and Long-Term Exposure of Cigarette Smoke Extract on Human Vocal Fold Fibroblasts. J Voice doi: 10.1016/j.jvoice.2023.08.002 37696688

[32] Martey CA, Pollock SJ, Turner CK, O’Reilly KM, Baglole CJ, Phipps RP, et al. (2004) Cigarette smoke induces cyclooxygenase-2 and microsomal prostaglandin E2 synthase in human lung fibroblasts: implications for lung inflammation and cancer. Am J Physiol Lung Cell Mol Physiol 287(5):L981–991. doi: 10.1152/ajplung.00239.2003 15234907

[33] Yang CM, Lee IT, Lin CC, Yang YL, Luo SF, Kou YR, et al. (2009) Cigarette smoke extract induces COX-2 expression via a PKCalpha/c-Src/EGFR, PDGFR/PI3K/Akt/NF-kappaB pathway and p300 in tracheal smooth muscle cells. Am J Physiol Lung Cell Mol Physiol 297(5):L892–902. doi: 10.1152/ajplung.00151.2009 19717552

[34] Lin CC, Lee IT, Yang YL, Lee CW, Kou YR, Yang CM (2010) Induction of COX-2/PGE(2)/IL-6 is crucial for cigarette smoke extract-induced airway inflammation: Role of TLR4-dependent NADPH oxidase activation. Free Radic Biol Med 48(2):240–254. doi: 10.1016/j.freeradbiomed.2009.10.047 19892012

[35] Wolf J, Weinberger B, Arnold CR, Maier AB, Westendorp RG, Grubeck-Loebenstein B (2012) The effect of chronological age on the inflammatory response of human fibroblasts. Exp Gerontol 47(9):749–753. doi: 10.1016/j.exger.2012.07.001 22790019 PMC3427851

[36] Nardini C, Moreau JF, Gensous N, Ravaioli F, Garagnani P, Bacalini MG (2018) The epigenetics of inflammaging: The contribution of age-related heterochromatin loss and locus-specific remodelling and the modulation by environmental stimuli. Semin Immunol 4049–60. doi: 10.1016/j.smim.2018.10.009 30396810

[37] Kabacik S, Horvath S, Cohen H, Raj K (2018) Epigenetic ageing is distinct from senescence-mediated ageing and is not prevented by telomerase expression. Aging (Albany NY) 10(10):2800–2815. doi: 10.18632/aging.101588 30332397 PMC6224244

[38] Shah K, McCormack CE, Bradbury NA (2014) Do you know the sex of your cells? Am J Physiol Cell Physiol 306(1):C3–18. doi: 10.1152/ajpcell.00281.2013 24196532 PMC3919971

[39] Luo SD, Chiu TJ, Chen WC, Wang CS (2021) Sex Differences in Otolaryngology: Focus on the Emerging Role of Estrogens in Inflammatory and Pro-Resolving Responses. Int J Mol Sci 22(16). doi: 10.3390/ijms22168768 34445474 PMC8395901

[40] Kirgezen T, Sunter AV, Yigit O, Huq GE (2017) Sex Hormone Receptor Expression in the Human Vocal Fold Subunits. J Voice 31(4):476–482. doi: 10.1016/j.jvoice.2016.11.005 27989395

[41] Voelter C, Kleinsasser N, Joa P, Nowack I, Martinez R, Hagen R, et al. (2008) Detection of hormone receptors in the human vocal fold. Eur Arch Otorhinolaryngol 265(10):1239–1244. doi: 10.1007/s00405-008-0632-x 18317786

[42] Newman SR, Butler J, Hammond EH, Gray SD (2000) Preliminary report on hormone receptors in the human vocal fold. J Voice 14(1):72–81. doi: 10.1016/s0892-1997(00)80096-x 10764118

[43] Schneider B, Cohen E, Stani J, Kolbus A, Rudas M, Horvat R, et al. (2007) Towards the expression of sex hormone receptors in the human vocal fold. J Voice 21(4):502–507. doi: 10.1016/j.jvoice.2006.01.002 16564673

[44] Mukudai S, Matsuda KI, Nishio T, Sugiyama Y, Bando H, Hirota R, et al. (2015) Differential responses to steroid hormones in fibroblasts from the vocal fold, trachea, and esophagus. Endocrinology 156(3):1000–1009. doi: 10.1210/en.2014-1605 25514085 PMC4330318

[45] Ozawa S, Mukudai S, Sugiyama Y, Branski RC, Hirano S (2021) Mechanisms Underlying the Antifibrotic Potential of Estradiol for Vocal Fold Fibrosis. Laryngoscope 131(10):2285–2291. doi: 10.1002/lary.29355 33378560

