# Supplementary figures and images for: Vocal fold fibroblasts and exposure to vibration in vitro: Does sex matter?
Source: PLoS One. 2024 Feb 9;19(2):e0297168. doi: 10.1371/journal.pone.0297168 (PMC10857603; doi:10.1371/journal.pone.0297168)

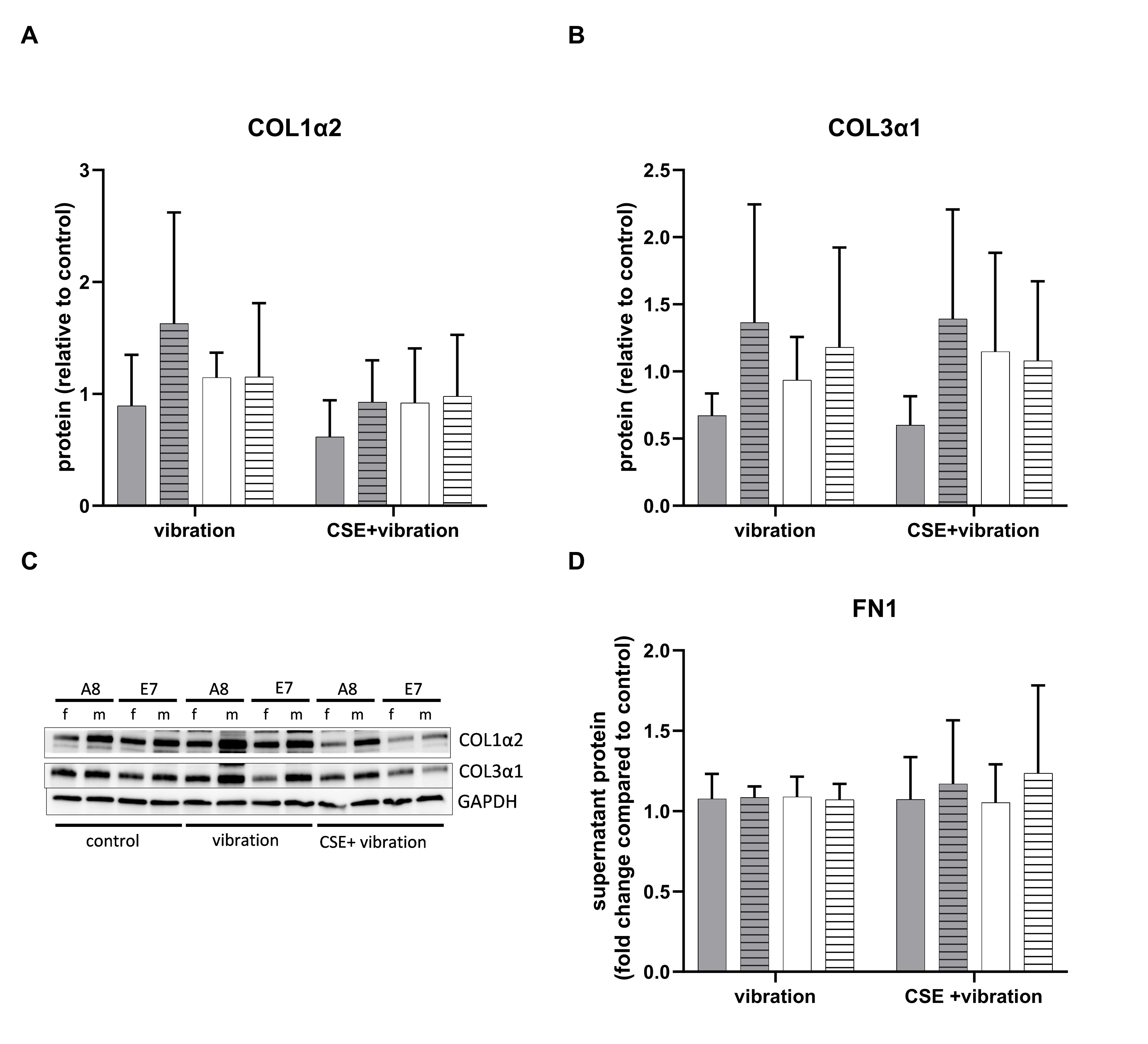

Supplement: S1 Fig — Protein levels of COL1A2 (A), COL3A1 (B), and FN1 (D) were analyzed by Western blot (A, B) and measured in the supernatant (D). Representative blots of COL1α2 and COL3α1 are shown in (C). All data are shown as mean and SD of fold changes compared to static control from six independent experiments (N = 6). Differences between the treatments within one vibration pattern and cell line were tested using RM one-way ANOVA with Šídák multiple comparison test or Friedman test with Dunn’s multiple comparisons test, depending on the result of the Shapiro-Wilk test of normality. Two-way ANOVA followed by Tukey’s multiple comparison test was used to compare the effect of different vibration patterns and cell lines. f = female vibration pattern, m = male vibration pattern. (TIF) [file pone.0297168.s001.tif]
