# Supplementary material for: Vocal fold fibroblasts and exposure to vibration in vitro: Does sex matter?
Source: PLoS One. 2024 Feb 9;19(2):e0297168. doi: 10.1371/journal.pone.0297168 (PMC10857603; doi:10.1371/journal.pone.0297168)
Supplement: S1 Raw images — (PDF) [file pone.0297168.s002.pdf]

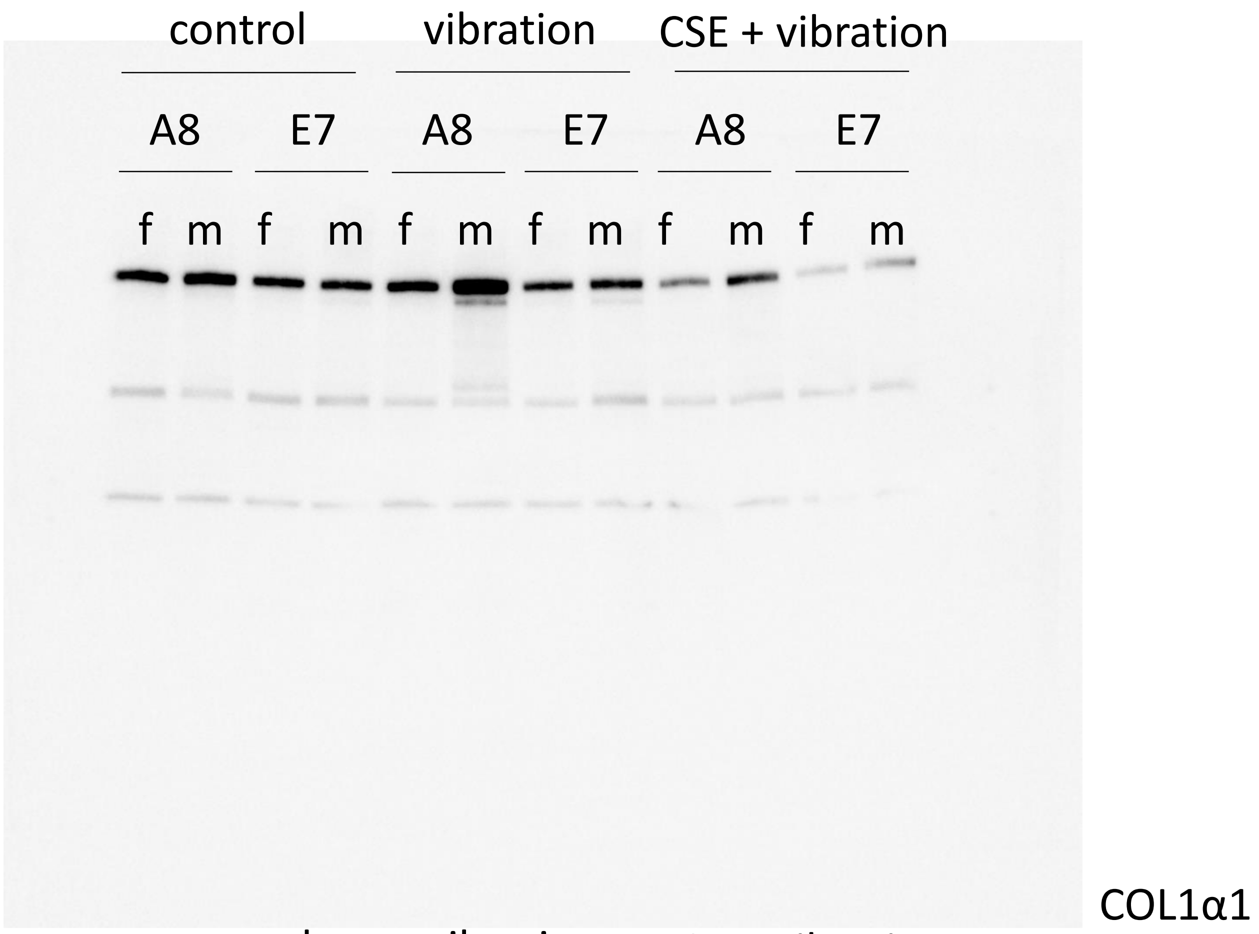

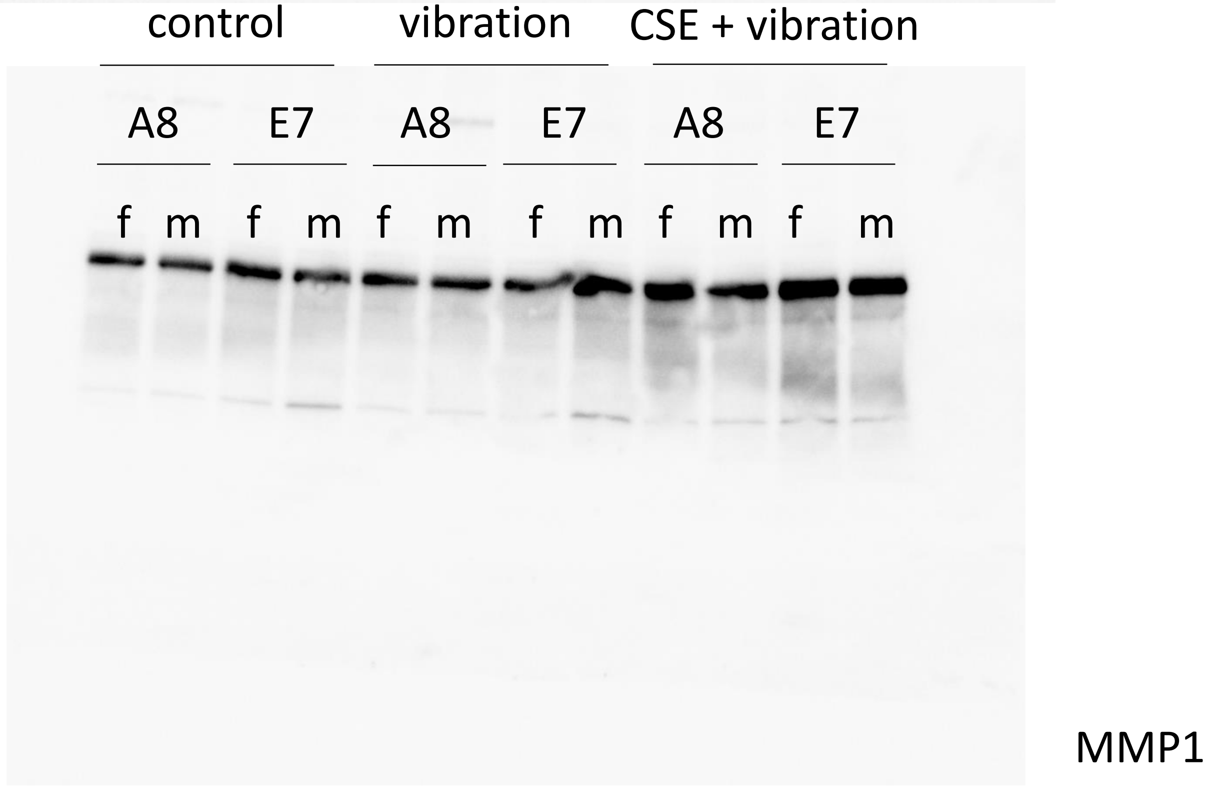

f = female pattern

m = male pattern

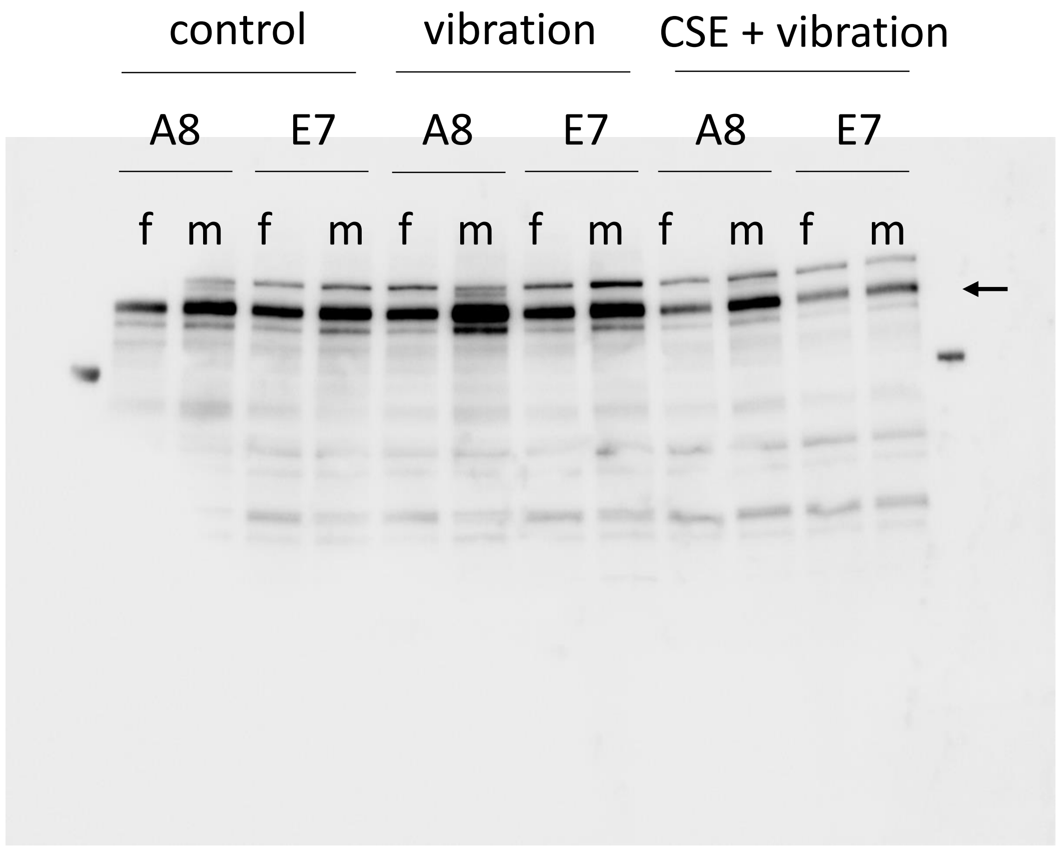

COL1α2

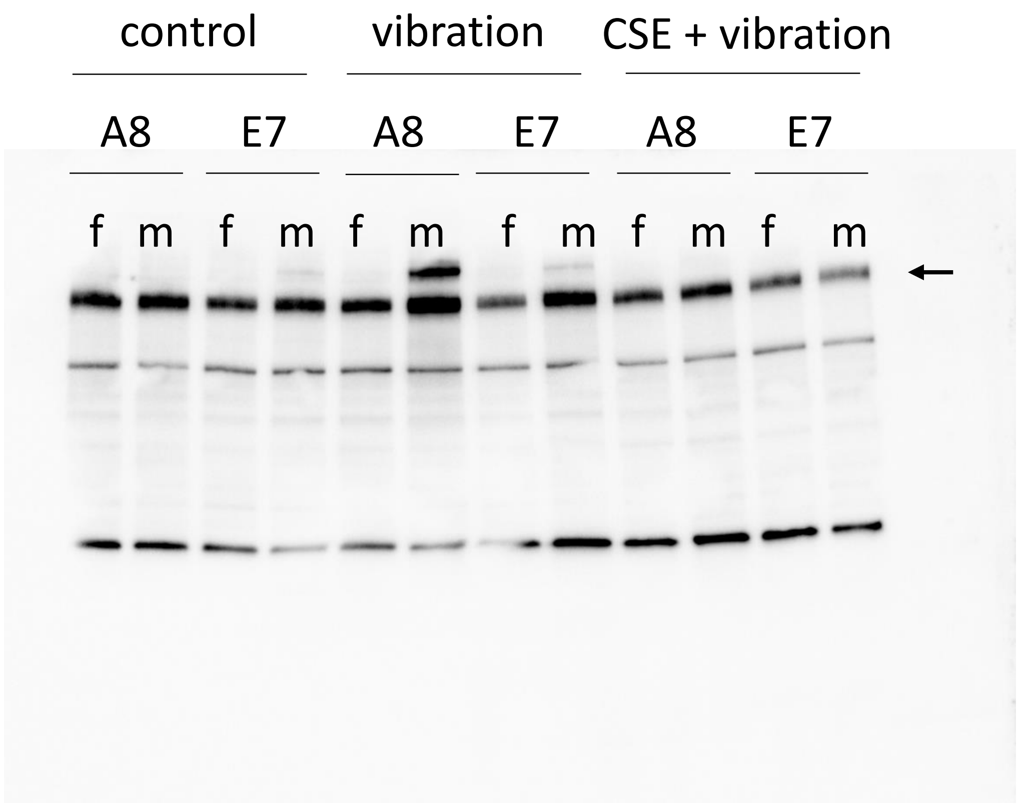

COL3α1

f = female pattern

m = male pattern

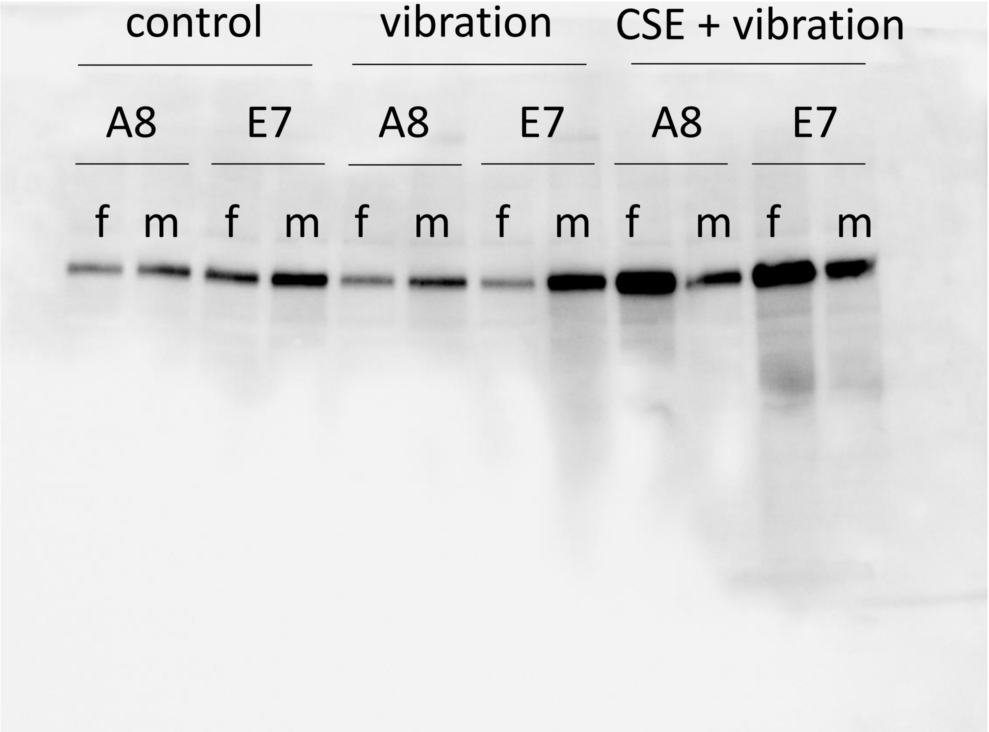

COX2

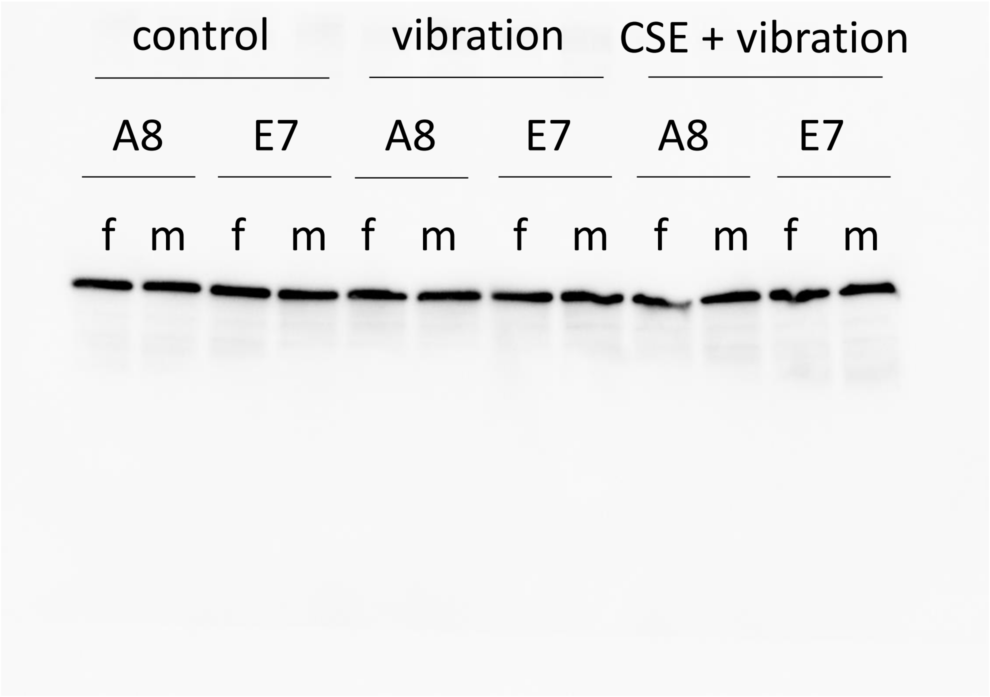

GAPDH

f = female pattern
m = male pattern
